# Menstrual Disturbances and Associated Factors Among Female University Students in the United Arab Emirates: A Cross-Sectional Study

**DOI:** 10.7759/cureus.82169

**Published:** 2025-04-13

**Authors:** Mazen Alhaj Ahmad, Nour Al Huda Al Khatib, Saif Alshehhi, Israa Farag, Alyazah Alsuwaidi, Meer Kadir, Amal Hussein, Bashair M Mussa

**Affiliations:** 1 College of Medicine, University of Sharjah, Sharjah, ARE; 2 Family and Community Medicine Department, University of Sharjah, Sharjah, ARE; 3 Basic Medical Science Department, University of Sharjah, Sharjah, ARE

**Keywords:** menstruation, menstruation disturbances, premenstrual dysphoric disorder, stress, university students

## Abstract

Background: Menstrual disturbances are prevalent among young females worldwide. However, the literature addressing this topic in the United Arab Emirates (UAE) is scarce. This study aimed to measure the prevalence of menstrual disturbances among female university students across the UAE and identify the most common practices and lifestyle factors associated with menstrual disturbances.

Methods: This cross-sectional study utilized convenience sampling and was distributed to female university students. The questionnaire was developed after a careful assessment of the literature and was conducted between February and March of 2023. The Diagnostic and Statistical Manual of Mental Disorders (DSM-5) criteria were used to evaluate the prevalence of premenstrual dysphoric disorder (PMDD) among the study population.

Results: Of the total 390 participants, 73.6% had at least one menstrual disturbance, with PMDD being the most common at 50.8%. Furthermore, PMDD was significantly associated with caffeine consumption (p = 0.047). Conditions such as oligomenorrhea (p = 0.005), hypomenorrhea (p = 0.021), and PMDD (p ≤ 0.001) were associated with higher stress scores. For managing menstrual disturbance, 71.5% did not seek professional advice, and only 7.7% of patients took prescribed medications. Most participants opted for self-management by bed rest (75.4%), consumption of tea and herbs (63.1%), and self-medication (54.4%).

Conclusion: The prevalence of menstrual problems among female university students in the UAE was 73.6%, which was high, with higher stress scores being correlated with multiple menstrual disturbances; thus, awareness programs and the provision of proper counseling are recommended to all universities across the UAE.

## Introduction

Menstruation is the cyclic process that women of reproductive age undergo, during which blood and endometrial tissue are discharged from the uterus through the vagina. The bleeding typically starts in adolescence, between the ages of 11 and 14, and occurs every 28 days on average. It lasts between two and seven days, resulting in an average blood loss of 20-80 ml [1,2].

Menstrual disturbances are among the most common gynecological conditions and are most prevalent among females between 20 and 24 years old [1]. Common menstrual disturbances include menstrual cycle irregularities, dysmenorrhea, polymenorrhea, oligomenorrhea, amenorrhea, menorrhagia, and premenstrual syndrome (PMS) [3]. Menstrual disturbances impose significant social, economic, and health implications on individuals, communities, and healthcare systems, with abnormal uterine bleeding treatment costing up to 1.55 billion dollars in the United States alone; however, they are not prioritized in public healthcare plans as they are viewed as minor health concerns [3-5].

Premenstrual dysphoric disorder (PMDD), which is known as the severe form of PMS, is a recognized psychiatric condition listed in the DSM-V, requiring the presence of at least five out of 11 specific symptoms for diagnosis [6]. Those affected by PMDD experience symptoms like irritability, depression, and excessive tension, which significantly affect their social and professional lives [7]. This disorder is linked to higher rates of suicidal thoughts and attempts and serious mental health issues and complications [8].

University life is a critical phase for students as it is associated with a wide range of stressful experiences that lead to significant changes in their healthy lifestyles due to external barriers [9,10]. Previous reports have shown that menstrual disturbances were associated with risk factors such as obesity, eating disorders, sleep disturbances, and perceived levels of stress [9,11-16]. Furthermore, menstrual disturbances contribute to significant academic and social life impairments, including absenteeism, difficulty in concentration, and lower academic achievement [17].

To our knowledge, there are few studies in the literature addressing the prevalence of menstrual disturbances among university students in the United Arab Emirates (UAE), with only dysmenorrhea and PMS being previously studied in the UAE population [18,19]. This data availability would be crucial in implementing new health services specifically targeting university students [3]. The objectives of this study are to evaluate the prevalence of menstrual disturbances among female university students across the UAE and to identify the most common practices and lifestyle factors that are associated with these disturbances.

This article was presented as a poster at the 2nd International Gynecology and Obstetrics Conference on March 4, 2023, in Abu Dhabi, UAE. It was also presented as an E-poster at the 6th Emirates Family Medicine Society Congress on March 16, 2023, in Dubai, UAE. Finally, it appeared as an oral presentation at the 10th Undergraduate Research Competition by Abu Dhabi University, Abu Dhabi, UAE.

## Materials and methods

Study population and design

This cross-sectional study was conducted using convenience sampling, with data collection being conducted between February and March of 2023. The study was approved by the Research Ethics Committee of the University of Sharjah (REC-22-02-14-06-S). A Participant Information Sheet (PIS) was provided at the beginning of the questionnaire, which stated that proceeding with the questionnaire indicates the agreement to participate in the study. Participants were informed of their right to withdraw from the study at any point prior to the submission of the questionnaire.

The inclusion criteria of this study were female university students residing in the UAE. Pregnant, lactating, or women using contraceptives in the last three months were excluded. Moreover, females who underwent hysterectomy or oophorectomy were also excluded.

Cochran’s formula $N = \frac{4P(1 - P)}{\text{ME}^2}$ was used to calculate the sample size in a cross-sectional study that aims to estimate a single proportion [20]. "N" is the sample size, "P" is the expected prevalence, which is estimated at 90% (P = 0.9) by a similar study conducted in Saudi Arabia, and "ME" is the marginal error [2]. The minimum sample size was calculated to be 144; however, it was increased by 10% to reduce any margin of error, resulting in a minimum sample size of 160.

Data collection tool

The instrument used in this study was an online self-administered questionnaire distributed among female university students across the UAE through social media platforms including WhatsApp, Instagram, Facebook, and X. Questionnaire items were developed after reviewing the literature, while questions pertaining to stress were retrieved from the PSS-4 [21] (see Appendix for the questionnaire). The questionnaire consisted of 32 items, divided into four main sections, which are participants’ demographics, characteristics of their menstrual cycle, practices during menstruation, and lifestyle behaviors, including diet, exercise frequency, and stress levels.

Body mass index (BMI) was calculated using the reported height and weight. Participants’ BMI values were categorized as underweight (BMI below 18.5 kg/m^2^), normal weight (BMI between 18.5 and 24.9 kg/m^2^), overweight (BMI between 25 and 29.9 kg/m^2^), or obese (BMI greater than 30 kg/m^2^) [22]. Overweight and obese categories were grouped into one (BMI greater than or equal to 25 kg/m^2^) due to the low percentage of obese participants.

Menstrual disturbances include polymenorrhea, which is when the menstrual cycle length is less than 21 days; oligomenorrhea, when the menstrual cycle length is more than 35 days; menorrhagia, when bleeding lasts more than seven days; and hypomenorrhe,a when bleeding lasts less than three days [1].

Based on the Diagnostic and Statistical Manual of Mental Disorders, Fifth Edition (DSM-5), PMDD diagnostic criteria state that, in most of the menstrual cycles, at least five symptoms (out of 11) must be experienced during the final week preceding menses, which start to improve within a few days after the onset of menses and become minimal or absent afterward [6].

Furthermore, one or more of the following symptoms should be experienced: marked affective lability (e.g., mood swings), irritability, depressed mood, or anxiety. Moreover, one or more of the following symptoms should be experienced: decreased interest in usual activities, subjective concentration difficulty, feeling lethargic, significant change in appetite, insomnia or hypersomnia, a sense of being overwhelmed, or physical symptoms such as breast, joint, or muscle pain [6].

These symptoms should be present in most cycles in the preceding year and are causing significant distress or interfering with usual activities. Finally, a provisional diagnosis was considered in this study as the diagnosis should be confirmed by prospective daily ratings, which was not possible due to the nature of the study [6].

The questionnaire was available in Arabic and English, as they are the most spoken languages in the UAE. It was pilot-tested before data collection to ensure reliability and validity. Filling out the questionnaire took approximately five to seven minutes. Anonymity was maintained to ensure the privacy of the participants.

Statistical analysis

The collected data were exported from Google Forms (Google LLC, Mountain View, California, United States) to Microsoft Excel (Microsoft Corp., Redmond, WA, United States) for initial processing, which included data cleaning and coding. The dataset was subsequently transferred to IBM SPSS Statistics for Windows, version 28.0 (released 2021, IBM Corp., Armonk, NY) for statistical analysis. Descriptive statistics were used for univariate analyses: categorical variables were expressed as frequencies and percentages, while scale variables were expressed as either means or medians. For bivariate analysis, Pearson’s Chi-square, Mann-Whitney, and Kruskal-Wallis tests were used to study relationships between menstrual disturbances and demographic and lifestyle factors. P-values less than 0.05 were considered statistically significant.

## Results

Demographic characteristics of the study population

The study questionnaire was completed by a total of 417 individuals, of whom 390 met the inclusion criteria. Of the total number of participants, 292 (74.9%) were non-UAE citizens, 204 (52.3%) were older than 20 years of age, and 385 (98.7%) were single. Table 1 summarizes the demographic characteristics of the study sample.

**Table 1:** Demographics of the study participants

Factors	n (%)
Nationality	
UAE citizens	98 (25.1%)
Non-UAE citizens	292 (74.9%)
Age	
<20 years	186 (47.7%)
>20 years	204 (52.3%)
Residence	
Sharjah	194 (49.7%)
Abu Dhabi	109 (27.9%)
Other Emirates	87 (22.3%)
Marital status	
Single	385 (98.7%)
Married	5 (1.3%)
BMI	
Underweight	52 (13.3%)
Normal	248 (63.6%)
Overweight/obese	87 (22.3%)
Missing values	3 (0.8%)

Menstrual patterns and disturbances

In our study, 294 (75.4%) participants reported having a regular menstrual cycle. Regarding cycle length, 321 (82.3%) respondents reported having a normal cycle length of 21-35 days, 28 (7.2%) respondents had polymenorrhea, and 41 (10.5%) respondents had oligomenorrhea. Furthermore, underweight, overweight, and obese participants were 2.19 times more likely to have oligomenorrhea compared to participants who had normal BMI (χ2 (1, N = 362) = 5.694, p = 0.017). Kruskal-Wallis test revealed a statistically significant difference in stress scores between participants of different cycle lengths, χ2(2, N = 390) = 10.4, p = 0.005. Stress scores were higher among participants with oligomenorrhea (Md = 10.5) in comparison to those with polymenorrhea (Md = 9) and those with normal cycle length (Md = 8).

Regarding the duration of menses, 333 (85.4%) participants had an average of three to seven days of bleeding per cycle, while 46 (11.8%) had menorrhagia and 11 (2.8%) had hypomenorrhea. a statistically significant difference in stress scores between individuals of different durations of menses (χ2(2, N = 390) = 7.77, p = 0.021). Stress scores were higher among participants with hypomenorrhea (Md = 10) in comparison to those with menorrhagia (Md = 9) and those with a normal duration of menses (Md = 8).

As for menarche, 342 (87.7%) participants reported a normal onset of menstruation between the ages of 10 and 14, 35 (9%) of them had their menarche after the age of 14, and 13 (3.3%) participants had their menarche before the age of 10. Of all participants, 57 (14.6%) reported missing at least one period in the last three months. As per the DSM-V criteria, 198 (50.8%) of the participants met the requirements for a provisional diagnosis of PMDD. The frequency of the symptoms is reported in Table 2.

**Table 2:** Premenstrual dysphoric disorder symptoms

Symptom	n	%
Criteria A		
Marked affective lability (e.g., mood swings, feeling suddenly sad or tearful, or increased sensitivity to rejection).	312	80%
Marked irritability or anger or increased interpersonal conflicts.	234	60%
Marked depressed mood, feelings of hopelessness, or self-deprecating thoughts.	281	72.1%
Marked anxiety, tension, and/or feelings of being keyed up or on edge.	198	50.8%
Criteria B		
Decreased interest in usual activities (e.g., work, school, friends, hobbies).	193	49.5%
Subjective difficulty in concentration.	154	39.5%
Lethargy, easy fatigability, or marked lack of energy.	276	70.8%
Marked change in appetite; overeating; or specific food cravings.	241	61.8%
Hypersomnia or insomnia.	200	51.3%
A sense of being overwhelmed or out of control.	213	54.6%
Physical symptoms such as breast tenderness or swelling, joint or muscle pain, a sensation of “bloating,” or weight gain.	285	73.1%

**Figure 1:**
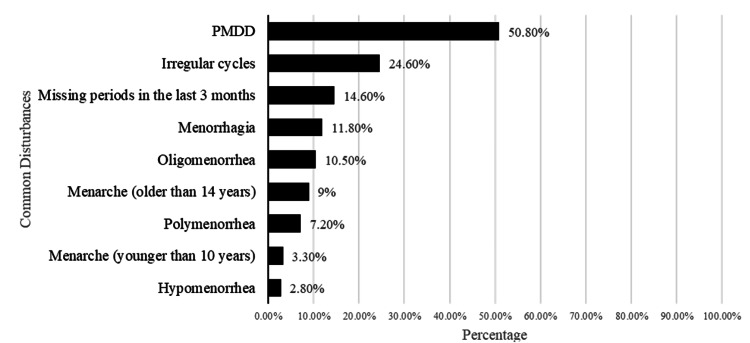
Distribution of the most common menstrual disturbances among study participants

A summary of the most common menstrual disturbances experienced by study participants is shown in Figure 1.

Caffeine drinking and stress were significantly associated with the prevalence of PMDD. Caffeine drinkers were 1.56 times more likely to have PMDD in comparison to non-drinkers (χ2 (1, N = 390) = 3.935, p = 0.047). In addition, stress scores were significantly higher among those who had PMDD (Md = 10, n = 198), compared to those with no PMDD (Md = 8, n = 192) (U = 13366, p ≤ 0.001) (Table 3).

**Table 3:** Factors correlated with prevalence of premenstrual dysphoric disorder (PMDD)

Factors	PMDD		P- value	Odds ratio (95% CI)
	Absent	Present		
Nationality				
UAE citizens (Ref)	46 (46.9%)	52 (53.1%)	0.600	0.885 (0.559-1.399)
Non-UAE citizens	146 (50.0%)	146 (50.0%)		
BMI				
Underweight	23 (44.2%)	29 (55.8%)	0.441	0.732 (0.401-1.335)
Normal (Ref)	129 (52.0%)	119 (48.0%)		
Overweight/Obese	40 (46.0%)	47 (54.0%)		1.274 (0.781-2.079)
Fast food intake				
No fast-food intake (Ref)	54 (55.7%)	43 (44.3%)	0.143	1.411 (0.889-2.239)
Fast food intake	138 (47.1%)	155 (52.9%)		
Caffeine intake				
No caffeine intake (Ref)	64 (57.1%)	48 (42.9%)	0.047	1.563 (1.004-2.432)
Caffeine intake	128 (46.0%)	150 (54.0%)		
Exercise				
Little/no exercise (Ref)	115 (50.2%)	114 (49.8%)	0.426	
1-3 times per week	51 (44.7%)	63 (55.3%)		1.246 (0.794-1.956)
+4 times per week	26 (55.3%)	21 (44.7%)		0.815 (0.434-1.531)

In this study, 287 (73.6%) of the participants had at least one menstrual disturbance, with 97 (24.9%) respondents having a positive family history of menstrual problems. However, only 57 (14.6%) participants had been previously diagnosed with menstrual problems.

Practices during menstruation

**Figure 2:**
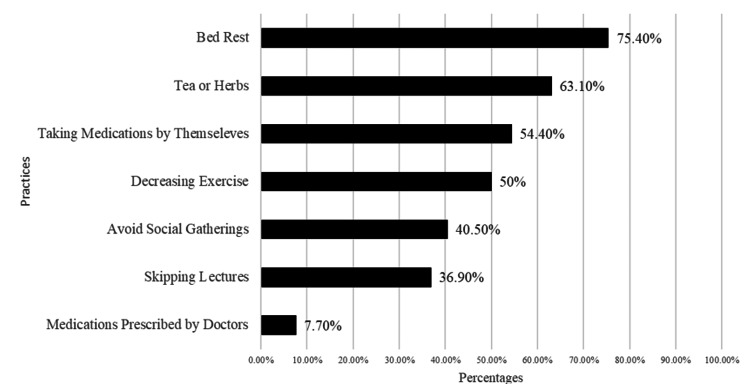
Distribution of the different practices done by the study participants during menstruation

As demonstrated in Figure 2, increased bed rest was the most commonly reported practice by participants during the period of menstruation, followed by increased intake of tea and herbs. Although more than half of the participants took medications during menses, only 30 (7.7%) respondents stated that they took medications following a doctor's prescription.

When inquired about seeking medical care for menstrual issues, 279 (71.5%) of the participants reported not consulting any physician. Among those who did seek medical attention, the majority visited gynecologists (91, 23.3%), followed by family physicians/general practitioners (33, 8.5%) and endocrinologists (19, 4.9%).

Twenty-seven (6.9%) participants followed specific diets such as “caloric deficit” and were 2.7 times more likely to have missed a period in the last three months compared to those who did not follow a specific diet (χ2 (1, N = 390) = 5.240, p = 0.022).

When asked about their weekly fast-food consumption, 205 (52.6%) participants reported consuming fast food one to two times per week, 88 (22.6%) respondents reported a consumption rate of more than three times a week, and 97 (24.9%) respondents reported not consuming fast food. Participants who consumed fast food were 2.68 times more likely to have oligomenorrhea (χ2 (1, N = 362) = 4.263, p = 0.039).

As for exercise, 229 (58.7%) participants stated that they performed little or no exercise. In terms of caffeine intake, 213 (54.6%) stated they had one to three caffeinated drinks per day, 65 (16.7%) had four or more drinks per day, and 112 (28.7%) denied drinking caffeinated drinks. The PSS-4 stress scale has a minimum score of 0 and a maximum score of 16. The median score of the participants was 9, with an interquartile range of 4. It is worth noting that no other statistically significant correlations were found between different menstrual disturbances and any demographic or lifestyle factors.

## Discussion

Menstrual disturbances are the most common gynecological complaints women experience that tend to impose significant challenges on females in the reproductive age [1]. University students are at particular risk of complications related to menstrual disturbances, such as diminished academic performance and decreased engagement in social and sports activities [17,23]. In this population in the UAE, the prevalence of dysmenorrhea and PMS has been previously studied by other researchers, but data on other prevalent menstrual disturbances remain scarce [18,19]. This study expands the scope of investigation to include understudied disorders such as PMDD and its association with different factors. 

This study found a high prevalence of menstrual disturbances, which was similarly reported in the literature [2,24-27]. Furthermore, 73.6% of the study sample suffered from at least one menstrual disturbance, while only 28.5% of the participants sought medical help for their problems. This explains the high prevalence rate of menstrual disturbances within the UAE, as not seeking medical help would not aid in treating potential underlying causes. This leads to an increase in symptomatic management and self-medication methods, which was observed in our results.

Not seeking medical help could be explained by several factors, including the students’ lack of awareness about menstrual health, the high cost of healthcare in the UAE, and the existing social stigma around the topic of menstruation in general [5,28]. This finding was also reported in a similar study conducted in Saudi Arabia, revealing that women preferred self-medicating over seeking professional help regardless of the severity of the symptoms [29].

Among participants, 24.6% had irregular menstrual cycles, which is similar to the rates found in Saudi Arabia (27%), higher than Egypt (12.5%), and lower than Lebanon (59.4%) [2,27,30]. Regarding the age of menarche, most participants had their first cycle between the ages of 10 and 14, which is in accordance with the previous reports [31,32].

The prevalence of menorrhagia was 11.8%, demonstrating a similar prevalence rate with Lebanon at 11.6%; furthermore, the prevalence in Saudi Arabia was lower at 9.1%, while the prevalence in Iran was higher at 19.2% [3,27,33]. Hypomenorrhea had a prevalence of 2.8%, similar to the findings in Lebanon (2%); furthermore, its prevalence was lower in comparison to both Saudi Arabia (6.7%) and Iran (5.25%) [3,27,33].

Regarding the menstrual cycle’s length, 10.5% of the participants had oligomenorrhea, which was higher than the rates reported in Saudi Arabia (5%) but lower than rates in both Lebanon (19.3%) and Iran (13.1%) [3,27,33]. Polymenorrhea was reported in 7.2% of the participants in this study, which was lower than the rates reported in Saudi Arabia (13%), Iran (9.9%), and Lebanon (37.5%) [3,27,33].

Although Saudi Arabia, UAE, Lebanon, and Iran are geographically proximal to each other, there is a significant difference in the prevalence of menstrual abnormalities. Thus, further research could be implemented to determine the factors leading to such alterations in prevalence and create targeted programs and awareness campaigns to manage such conditions properly.

This study found a correlation between fast food intake and oligomenorrhea, which is consistent with the findings in India [34]. This association could be attributed to the high trans-fat and refined sugar content of fast foods, which could disturb endocrine functions and menstrual regularity. However, our study did not observe any correlations between fast food intake and other menstrual disturbances, which could be explained by differences in the nature and habits of fast-food consumption in both countries. In addition, we have found that abnormal BMI was correlated with oligomenorrhea, which might be explained by the fact that higher obesity levels were associated with increased incidence of menstrual disturbances such as oligomenorrhea and amenorrhea [35].

The correlation between following specific diets and missing a menstrual cycle in the previous three months can be attributed to chronic energy deficiency leading to abnormalities in the hypothalamic-pituitary-gonadal axis; thus, it would lead to a suppression of reproductive function due to functional hypothalamic amenorrhea (FHA) [36].

PMDD is defined as the recurrence of affective symptoms that are distressing and impairing in a cyclical manner [37]. Women suffering from PMDD experience certain characteristic features such as mood lability, irritability, and anxiety symptoms, all of which can be accompanied by other physical and behavioral symptoms. These symptoms must be present a few days before the onset of menses (DSM-V) [6]. The rate of PMDD found in this study was 50.8%, which is much higher than the estimated global rate (3-8%); additionally, it was also higher than the rates reported in Saudi Arabia (12.5%), Egypt (21.1%), and Ethiopia (37.4%) [7,8,38,39].

Multiple psychiatric disorders, such as anxiety and mood disorders, exhibit overlapping symptoms with PMDD. This overlap can potentially lead to misdiagnosis, contributing to elevated PMDD prevalence. The high rates of anxiety (55%) and depression (38%), as observed in a study conducted among UAE university students, might explain the high PMDD prevalence found in our study [40]. Additionally, as stated by the DSM-V, major depressive disorder (MDD) is the most common comorbid disorder among women suffering from PMDD; therefore, the potential of having other comorbidities concurrent with PMDD sharing similar symptoms makes an accurate diagnosis of PMDD difficult and may explain the high prevalence found [41].

There are multiple factors influencing the incidence and characteristics of PMDD, including different environmental (e.g., stress and trauma), genetic, hormonal, and reproductive factors, all of which influence the hypothalamic-pituitary-adrenal (HPA) axis [42]. In this study, PMDD was associated with caffeine intake, which was found in previous studies that additionally reported an association between PMDD prevalence and consumption of sweetened drinks and tea and increased food intake under stress [43,44]. This association could be attributed to the antagonistic effect of caffeine on adenosine receptors, disturbing serotonin neurotransmission, which possibly intensifies PMDD affective symptoms [45]. The discrepancy between the high PMDD identified in this study (50.8%) and lower global prevalence rates (2-3%) may be explained by two concurrent phenomena: (1) potential overestimation due to sharing diagnostic criteria with other psychiatric disorders [41] and (2) persistent underdiagnosis in clinical settings resulting from societal stigma and lack of awareness [46]. This underscores the need for both improved diagnostic accuracy through prospective daily symptom tracking of PMDD and community-based interventions to reduce healthcare-seeking barriers.

We recommend that future researchers repeat this study on a broader scale and include females from different demographics and socioeconomic backgrounds to further enhance the accuracy of the findings. Furthermore, since our study included various menstrual disturbances that have not been previously studied in the UAE, we recommend that future studies explore additional associations between specific menstrual disturbances and other factors that have not been explored within this study's scope, such as academic burden, socioeconomic status and sleep behaviour. Finally, given the retrospective nature of this study, there is a potential for recall bias, introducing a risk that the rates could be skewed. Therefore, we strongly recommend that other researchers studying PMDD prevalence provide a questionnaire with daily ratings for accurate PMDD diagnosis [6].

Limitations of the study

The data were collected through a self-reported questionnaire, which is subject to bias; therefore, further studies with more comprehensive elements are warranted. The most common attitudes during menstruation were categorized into a list that participants selected from; therefore, it might not be inclusive of all attitudes practiced by participants. Due to the study’s cross-sectional design, it was not possible to infer causation between some possible risk factors and menstrual disturbances. Moreover, it was not possible to confirm the diagnosis of PMDD due to the requirement of prospective daily ratings of symptoms, which was not possible to obtain due to the study design.

## Conclusions

This study highlights the high prevalence of menstrual disturbances among female university students in the UAE, with conditions such as PMDD, irregular cycles, and abnormal bleeding patterns being common. Despite experiencing symptoms, a significant number of participants did not seek medical care, likely due to a lack of awareness. In addition, lifestyle factors such as diet, stress, and physical activity were found to be associated with menstrual irregularities. The strong presence of PMDD also underscores the need for better mental health support within university settings.

To address these issues, we recommend implementing targeted awareness programs to educate students on menstrual health and its impact and establishing accessible gynecological and psychological services within universities that could improve early diagnosis and management. Future studies should explore the long-term effects of lifestyle factors on menstrual health and assess the effectiveness of intervention programs in promoting better health outcomes.

## Appendices

Questionnaire

**Table 4:** English version of the questionnaire This questionnaire was developed by the authors of this study after reviewing the literature about the topic “Credits for developing the questionnaire: Mazen Alhaj Ahmad, Nour Al Huda Al Khatib, Saif Alshehhi, Israa Farag, Alyazah Alsuwaidi, Meer Kadir. Reviewed for scientific accuracy: Bashair M Mussa”

Item number (Section name)	Question	Answers
A1 (Demographics)	What is your sex?	Female
		Male
A2	Do you live in the UAE?	Yes
		No
A3	Are you a university student?	Yes
		No
A4	How old are you?	Less than 20 years old
		Between 20 and 30 years old
		More than 30 years old
A5	What is your nationality?	Local (Emirati)
		Other Arab (Non-Emirati)
		Non Arab
A6	Which emirate do you live in?	Sharjah
		Abu Dhabi
		Dubai
		Ajman
		Ras Al Khaimah
		Fujairah
		Umm Al Quwain
A7	What is your Weight? (in kg)	Open-ended question
A8	What is your Height? (in cm)	Open-ended question
A9	What is your marital status?	Single
		Married
		Divorced/Widow
A10	Are you currently pregnant or breastfeeding? (If married)	Yes
		No
A11	Did you take any oral contraceptive pills in the last 3 months? (If married)	Yes
		No
A12	Did you undergo uterus/ovaries removal surgery?	Yes
		No
B1 (Characteristics of menstrual cycles)	How old were you when you got your first menstruation?	Less than 10 years old
		Between 10 and 14 years old
		More than 14 years old
B2	Describe the regularity of your menstrual cycle in the last 12 months?	Regular
		Irregular
B3	What is the average time between 2 periods?	Less than 21 days
		Between 21-35 days
		More than 35 days
B4	What is the average duration of your period?	2 days or less
		Between 3-7 days
		8 days or more
B5	Did you miss your periods (all three if regular every 28 days) in the last 3 months?	Yes
		No
B6	In the 10 days before menstruation (most cycles for the last year, did you experience any of the following? (Select all that apply)	Mood swings
		Anger
		Depressed mood
		Anxiety
		Decreased interest in usual activities
		Difficulty in concentration
		Lack of energy
		Change in appetite
		Decreased sleep or Increased sleep
		Feeling overwhelmed
		Physical Symptoms (Breast tenderness/swelling, joint or muscle pain, feeling bloated, weight gain)
C1 (Attitudes)	Do you practice any of the following behaviors during your period?(Select all that apply)	Medications prescribed by doctors
		Taking medications by yourself
		Tea or herbs
		Bed rest
		Skipping lectures
		Decreasing your exercise
		Avoid social gatherings
C2	Have you ever visited any of the following for any problems related to your period? (Select all that apply)	Gynecologist
		Endocrinologist
		Family Doctor/ General practitioner
		I didn't visit any doctor
C3	Were you diagnosed with a menstrual-related problem previously?	Yes
		No
C4	Does any member of your family suffer from menstrual problems?	Yes
		No
D1 (Lifestyle)	How many times per week do you eat fast food?	I usually don't eat fast food
		1 or 2 times per week
		3 times per week or more
D2	Do you follow any specific diet?	Open-ended question
D3	How often do you drink Caffeinated drinks (Tea, coffee, energy, drinks)?	I don't usually drink caffeinated drinks
		1 to 3 times per day
		More than 4 times per day
D4	How often do you exercise weekly?	little or no exercise
		1-3 times/week
		4-5 times/week
		daily exercise or intense exercise 3-4 times/ week
		intense exercise 6-7 times /week
		very intense exercise daily or physical job
D5 (Lifestyle- stress)	In the last month, how often have you felt that you were unable to control the important things in your life?	1 (Never)
		2
		3
		4
		5 (Very Often)
D6	In the last month, how often have you felt confident about your ability to handle your personal problems?	1 (Never)
		2
		3
		4
		5 (Very Often)
D7	In the last month, how often have you felt that things were going your way?	1 (Never)
		2
		3
		4
		5 (Very Often)
D8	In the last month, how often have you felt difficulties were piling up so high that you could not overcome them?	1 (Never)
		2
		3
		4
		5 (Very Often)
